# Eliminating mother-to-child transmission of human immunodeficiency virus, syphilis and hepatitis B in sub-Saharan Africa

**DOI:** 10.2471/BLT.20.272559

**Published:** 2021-01-21

**Authors:** Jennifer Cohn, Morkor N Owiredu, Melanie M Taylor, Philippa Easterbrook, Olufunmilayo Lesi, Bigirimana Francoise, Laura N Broyles, Angela Mushavi, Judith Van Holten, Catherine Ngugi, Fuqiang Cui, Dalila Zachary, Sirak Hailu, Fatima Tsiouris, Monique Andersson, Dorothy Mbori-Ngacha, Wame Jallow, Shaffiq Essajee, Anna L Ross, Rebecca Bailey, Jesal Shah, Meg M Doherty

**Affiliations:** a University of Pennsylvania School of Medicine, Division of Infectious Diseases, 3400 Spruce St, Philadelphia, PA 19104, United States of America (USA).; b Global HIV, Hepatitis and STI Programmes, World Health Organization, Geneva, Switzerland.; c World Health Organization, Regional Office for Africa, Brazzaville, Republic of the Congo.; d Global Health Impact Group, Atlanta, USA.; e Ministry of Health of Zimbabwe, Harare, Zimbabwe.; f Ministry of Health of Kenya, Nairobi, Kenya.; g The Global Fund, Geneva, Switzerland.; h Department of Maternal, Newborn, Child and Adolescent Health, World Health Organization, Windhoek, Namibia.; i ICAP at Columbia University, New York, USA.; j Division of Medical Virology, Stellenbosch University, Tygerberg, South Africa.; k HIV Section, United Nations Children’s Fund, New York, USA.; l International Treatment Preparedness Coalition, Gaborone, Botswana.; m Science Division, World Health Organization, Geneva, Switzerland.; n Elizabeth Glaser Pediatric AIDS Foundation, Geneva, Switzerland.

## Abstract

Triple elimination is an initiative supporting the elimination of mother-to-child transmission of three diseases – human immunodeficiency virus (HIV) infection, syphilis and hepatitis B. Significant progress towards triple elimination has been made in some regions, but progress has been slow in sub-Saharan Africa, the region with the highest burden of these diseases. The shared features of the three diseases, including their epidemiology, disease interactions and core interventions for tackling them, enable an integrated health-systems approach for elimination of mother-to-child transmission. Current barriers to triple elimination in sub-Saharan Africa include a lack of policies, strategies and resources to support the uptake of well established preventive and treatment interventions. While much can be achieved with existing tools, the development of new products and models of care, as well as a prioritized research agenda, are needed to accelerate progress on triple elimination in sub-Saharan Africa. In this paper we aim to show that health systems working together with communities in sub-Saharan Africa could deliver rapid and sustainable results towards the elimination of mother-to-child transmission of all three diseases. However, stronger political support, expansion of evidence-based interventions and better use of funding streams are needed to improve efficiency and build on the successes in prevention of mother-to-child transmission of HIV. Triple elimination is a strategic opportunity to reduce the morbidity and mortality from HIV infection, syphilis and hepatitis B for mothers and their infants within the context of universal health coverage.

## Résumé

La triple élimination est une initiative visant à soutenir l'éradication de la transmission mère-enfant de trois maladies – l'infection au virus de l'immunodéficience humaine (VIH), la syphilis et l'hépatite B. Bien que des avancées considérables aient été observées en ce sens dans certaines régions, les progrès demeurent lents en Afrique subsaharienne, pourtant durement touchée par ces maladies. Les caractéristiques communes aux trois affections, notamment leur épidémiologie, les interactions entre elles et les principales interventions nécessaires à leur prise en charge permettent aux systèmes de santé d'adopter une approche intégrée pour éviter la transmission mère-enfant. Plusieurs obstacles entravent actuellement la triple élimination en Afrique subsaharienne, parmi lesquels l'absence de politiques, de stratégies et de ressources pour garantir la disponibilité de traitements préventifs et curatifs bien établis. Les outils existants offrent déjà de nombreuses solutions; mais pour accélérer la progression de cette triple élimination en Afrique subsaharienne, il est indispensable de développer de nouveaux produits et modèles de soins, ainsi qu'un programme de recherche prioritaire. Dans le présent document, nous voulons montrer que si les systèmes de santé collaborent avec les communautés en Afrique subsaharienne, ils pourront obtenir des résultats rapides et durables en vue d'éradiquer la transmission mère-enfant des trois maladies susmentionnées. Néanmoins, une telle démarche implique un soutien politique massif, l'expansion des interventions fondées sur des données scientifiques, et une meilleure utilisation des sources de financement afin d'améliorer l'efficacité et de s'appuyer sur les réussites en matière de prévention de la transmission du VIH de la mère à l'enfant. La triple élimination représente une occasion stratégique de réduire la morbidité et la mortalité liées à l'infection au VIH, à la syphilis et à l'hépatite B, tant chez les mères que chez les nourrissons, dans un contexte de couverture maladie universelle.

## Resumen

La triple eliminación es una iniciativa que apoya la eliminación de la transmisión maternoinfantil de tres enfermedades: la infección por el virus de la inmunodeficiencia humana (VIH), la sífilis y la hepatitis B. En algunas regiones se han logrado avances significativos hacia la triple eliminación, pero los progresos se han desarrollado con mayor lentitud en el África subsahariana, la región con la mayor carga de estas enfermedades. Las características comunes de las tres enfermedades, como su epidemiología, las interacciones entre ellas y las intervenciones básicas para combatirlas, permiten un enfoque integrado de los sistemas de salud para la eliminación de la transmisión maternoinfantil. Los obstáculos actuales para la triple eliminación en el África subsahariana incluyen la falta de políticas, estrategias y recursos para apoyar la adopción de intervenciones preventivas y de tratamiento bien establecidas. Aunque se puede lograr mucho con las herramientas existentes, se necesita el desarrollo de nuevos productos y modelos de atención, así como una agenda de investigación prioritaria, para acelerar el progreso de la triple eliminación en el África subsahariana. En este documento pretendemos demostrar que los sistemas de salud que trabajan conjuntamente con las comunidades del África subsahariana podrían obtener resultados rápidos y sostenibles hacia la eliminación de la transmisión maternoinfantil de las tres enfermedades. Sin embargo, se necesita un mayor apoyo político, la ampliación de las intervenciones basadas en la evidencia y un mejor uso de los flujos de financiación para mejorar la eficiencia y aprovechar los éxitos en la prevención de la transmisión maternoinfantil del VIH. La triple eliminación es una oportunidad estratégica para reducir la morbilidad y la mortalidad de la infección por el VIH, la sífilis y la hepatitis B para las madres y sus hijos en el contexto de la cobertura sanitaria universal.

## ملخص

إن القضاء على هذه الأمراض الثلاثة هو مبادرة لدعم القضاء على انتقال ثلاثة أمراض من الأم إلى الطفل، وهي عدوى فيروس نقص المناعة البشرية (HIV)، والزهري، والالتهاب الكبدي ب. تم إحراز تقدم كبير نحو القضاء على هذه الأمراض الثلاثة في ​​بعض المناطق، إلا أن التقدم المحرز كان بطيئًا في جنوب الصحراء الكبرى بأفريقيا، وهي المنطقة التي تزخر بأكبر عبء لهذه الأمراض. إن السمات المشتركة لهذه الأمراض الثلاثة، بما يشمل سماتها في علم الأوبئة، وتفاعلاتها المرضية والتدخلات الأساسية للتعامل معها، تتيح اتباع نهج النظم الصحية المتكاملة للقضاء على انتقال العدوى من الأم إلى الطفل. إن العوائق الحالية أمام القضاء على الأمراض الثلاثة في جنوب الصحراء الكبرى بأفريقيا، تشمل الافتقار إلى السياسات والاستراتيجيات والموارد لدعم استيعاب التدخلات الوقائية والعلاجية الراسخة. بينما يمكن إنجاز الكثير باستخدام الأدوات الحالية، إلا أن هناك حاجة إلى تطوير منتجات ونماذج جديدة للرعاية، وكذلك وضع جدول أعمال بحثي على رأس الأولويات، لتسريع التقدم في القضاء على الأمراض الثلاثة في جنوب الصحراء الكبرى بأفريقيا. نهدف في هذه الورقة البحثية إلى توضيح أن الأنظمة الصحية التي تعمل جنبًا إلى جنب مع المجتمعات في جنوب الصحراء الكبرى بأفريقيا، يمكنها أن تحقق نتائج سريعة ومستدامة في سبيل القضاء على انتقال الأمراض الثلاثة من الأم إلى الطفل. وبالرغم من ذلك، فإن هناك حاجة إلى دعم سياسي أقوى، وتوسيع نطاق التدخلات المعتمدة على الدلائل، واستخدام أفضل لتدفقات التمويل، وذلك لتحسين الكفاءة، واستغلال النجاحات في الحيلولة دون انتقال فيروس نقص المناعة البشرية من الأم إلى الطفل. إن القضاء على الأمراض الثلاثة هو فرصة استراتيجية للحد من الحالات المرضية والوفيات من جراء عدوى فيروس نقص المناعة البشرية والزهري والالتهاب الكبدي ب، لكل من الأمهات ومواليدهن في سياق التغطية الصحية الشاملة.

## 摘要

三重消除是一项支持消除三种疾病【人类免疫缺陷病毒 (HIV) 感染、梅毒和乙型肝炎】母婴传播的计划。在有些地区，三重消除取得了重大进展，但在撒哈拉以南非洲地区，该计划的进展缓慢，因此，该地区在对抗这些疾病方面的任务最为繁重。这三种疾病在其流行病学、疾病相互作用和相关核心干预措施方面具有共同特点，因此，卫生系统能够综合采取方法来消除母婴传播。目前阻碍撒哈拉以南非洲地区三重消除计划实施的障碍包括缺乏支持采用既定预防和治疗措施的政策、战略和资源。虽然利用现有工具可取得很大的成就，但还是需要开发新的护理产品和模式，并制定优先研究议程，以加快撒哈拉以南非洲地区三重消除计划的进展。在本文中，我们的目的在于表明撒哈拉以南非洲地区的卫生系统与社区开展合作，能够为消除这三种疾病的母婴传播带来迅速且可持续的效果。但是，需要加强政治支持、扩大循证干预措施和更好地利用资金流，以提高效率，并在预防 HIV 母婴传播方面奠定成功的基础。三重消除是一种战略机遇，可以在全民保健范围内降低母亲及其婴儿因感染 HIV、梅毒和乙型肝炎而导致的发病率和死亡率。

## Резюме

Тройная элиминация, это инициатива, поддерживающая элиминацию передачи от матери ребенку трех заболеваний: инфекции, вызванной вирусом иммунодефицита человека (ВИЧ), сифилиса и гепатита В. В некоторых регионах был достигнут значительный прогресс в направлении тройной элиминации, однако на территориях к югу от Сахары (в регионе Африки с самым высоким бременем этих болезней) такой прогресс был медленным. Общие характеристики трех болезней, включая их эпидемиологию, взаимодействие болезней и основные меры по борьбе с ними, позволяют использовать комплексный подход в рамках систем здравоохранения к элиминации передачи от матери ребенку. Текущие препятствия на пути тройной элиминации в странах Африки к югу от Сахары включают отсутствие политик, стратегий и ресурсов для поддержки внедрения хорошо зарекомендовавших себя профилактических и лечебных мер. Несмотря на то что с помощью существующих инструментов можно добиться многого, для ускорения прогресса в области тройной элиминации в странах Африки к югу от Сахары необходима разработка новых продуктов и моделей оказания медицинской помощи, а также повестка дня в области приоритетных научных исследований. В этом документе авторы стремятся показать, что системы здравоохранения, работающие вместе с общественностью в странах Африки к югу от Сахары, могут обеспечить быстрые и устойчивые результаты в направлении элиминации передачи всех трех заболеваний от матери ребенку. Однако для повышения эффективности и достижения успехов в профилактике передачи ВИЧ от матери ребенку необходимы: более сильная политическая поддержка, расширение научно обоснованных мер и лучшее использование потоков финансирования. Тройная элиминация, это стратегическая возможность снизить заболеваемость и смертность от ВИЧ-инфекции, сифилиса и гепатита B для матерей и их младенцев в контексте всеобщего охвата услугами здравоохранения.

## Triple elimination

Triple elimination is the elimination of mother-to-child transmission of three infections prevalent in low- and middle-income countries – human immunodeficiency virus (HIV), syphilis and hepatitis B virus. The African Region of the World Health Organization (WHO) has the highest burden of the three infections (Table 1). While other regions have made progress in establishing and/or implementing policies or interventions to reduce mother-to-child transmission of these infections, the African Region is trailing and has the greatest need for improvements in service coverage to reach triple elimination. The current coronavirus disease 2019 (COVID-19) pandemic has disrupted health services and access to care across the world. Services related to triple elimination such as antenatal care and routine immunization have been disrupted in 56% (58/104) and 61% (64/105) of countries, respectively.^6^ Access to HIV care and antiretroviral treatment has been disrupted in 32% (32/101) of countries. These disruptions are likely to reduce gains made in triple elimination globally, especially in sub-Saharan Africa.^6^^,^^7^

**Table 1:** Prevalence of HIV, hepatitis B and syphilis in World Health Organization regions

Infection	Estimate of prevalence, %, by WHO region					
	African	American	South-East Asia	European	Eastern Mediterranean	Western Pacific
HIV, age 15–49 years	1.4–6.7	0.2–1.1	0.3	0.2–0.9	< 0.1	0.1
Syphilis in pregnant women	1.5	0.9	0.2	0.1	0.8	0.3
Hepatitis B, children < 5 years	3.0	0.2	0.7	0.4	0.5	0.9

HIV: human immunodeficiency virus; WHO: World Health Organization.

Sources: UNAIDS,^1^ Korenromp et al.,^2^ WHO.^3^^–^^5^

The rationale for a linked agenda for these three diseases is because they share several common features. First, a shared burden: all three diseases are highly prevalent and cause considerable morbidity and mortality in sub-Saharan Africa.^1^^,^^2^^,^^8^^,^^9^ Second, the importance of mother-to-child transmission: mother-to-child transmission rates in the absence of interventions range from 15% to 41% for HIV, 30% to 100% for syphilis and 10% to 90% for hepatitis B virus.^1^^,^^2^^,^^8^^,^^9^ Third, their synergistic effect: HIV interacts with both syphilis and hepatitis B virus to increase mother-to-child transmission risk.^9^^–^^11^ Coinfection with HIV and hepatitis B virus results in higher maternal hepatitis B virus deoxyribonucleic acid (DNA) levels and greater mother-to-child transmission of hepatitis B virus compared with infection only with hepatitis B virus.^9^ Children of mothers with both HIV infection and syphilis are at significantly increased risk for intrauterine HIV transmission compared with children of mothers infected only with HIV.^10^ Adverse birth outcomes are more common in babies of women with both HIV infection and syphilis than in babies of women with only one of these infections.^11^ Fourth, their easy diagnosis: all three infections can be readily diagnosed with rapid blood-based diagnostic tests. Fifth, prevention: mother-to-child transmission of all three infections can be prevented with interventions specific to each infection. Maternal interventions include: (i) initiation of lifelong antiretroviral therapy in HIV-infected mothers; (ii) a single dose of benzathine penicillin administered to pregnant women with syphilis at least 30 days before delivery; and (iii) antiviral prophylaxis with tenofovir in the third trimester of pregnancy for mothers with hepatitis B who have hepatitis B virus early antigen (HBeAg) or high viral load. Interventions for the baby further reduce the risk of infection; these interventions include antiretroviral prophylaxis for the first 6–12 weeks of life for HIV-exposed infants, and provision of hepatitis B immunoglobulin for babies born to mothers with hepatitis B where there is a high risk of transmission (Table 2). For hepatitis B virus, the most important intervention to prevent mother-to-child transmission is hepatitis B vaccination. All infants should receive a birth dose of hepatitis B virus monovalent vaccine followed by two or three additional doses during the first year. For all three infections, reproductive, maternal, neonatal and child health services provide a common entry point for the delivery of interventions.^13^^,^^14^

**Table 2:** Mother-to-child transmission of HIV, hepatitis B and syphilis in sub-Saharan Africa: epidemiology, diagnosis and interventions

Parameter	HIV	Syphilis	Hepatitis B
Epidemiology	25.6 million people living with HIV (prevalence of 1.5% in west and central Africa, 7% east and southern Africa) 440 000 deaths in 2019 120 000 new infections in children in 2019 87 000 deaths in children in 2019	564 000 pregnant women with active syphilis (prevalence 1.52%) in 2016 404 000 cases of congenital syphilis annually, 216 000 adverse birth outcomes, including death, in 2016	About 60 million HBsAg-positive (6.1% prevalence) in 2015 90 000 deaths annually in 2016 About 3% prevalence in 5-year-olds in 2015 360 000 new infections in infants in 2016
Diagnostics	Routine screening with HIV rapid diagnostic test (as early as possible in pregnancy, with retesting in third trimester in settings with a high prevalence of HIV); consider retesting during breastfeeding	Syphilis rapid diagnostic test (at the first antenatal care visit or as early as possible thereafter)	Routine screening with HBsAg rapid test (as early as possible in pregnancy)
Interventions to prevent mother-to-child transmission	Maternal antiretroviral therapy for life; antiretroviral therapy as infant prophylaxis for 6–12 weeks	Maternal treatment with penicillin	Hepatitis B vaccine at birth plus three additional doses in the first year of life; hepatitis B immunoglobulin for exposed infants; hepatitis B antiviral treatment for women with high hepatitis B virus DNA load
Coverage of key interventions	83% of pregnant women received antiretroviral therapy in 2019 (60% in west and central Africa, 91% east and southern Africa)	47% of pregnant women screened for syphilis in 2016 76% of pregnant women treated for syphilis in 2016	4% of infants received hepatitis B birth dose vaccine coverage in 2018 Inadequate hepatitis B screening

DNA: deoxyribonucleic acid; HIV: human immunodeficiency virus; HBsAg: hepatitis B surface antigen.

Sources: UNAIDS,^1^ Korenromp et al.,^2^ and World Health Organization.^3^^,^^12^

Political and technical frameworks provide tools and targets to support triple elimination. In 2016, WHO created three interlinked global health sector strategies on HIV, viral hepatitis and sexually transmitted infections.^15^^–^^17^ The Organization provided definitions, guidance and a validation process for the dual elimination of mother-to-child transmission of HIV and syphilis, and is planning to add hepatitis B virus for a triple elimination initiative.^18^ The 2016 WHO global health sector strategy on viral hepatitis set a target for the prevalence of hepatitis B surface antigen (HBsAg) in children aged 5 years of less than 0.1% for elimination of hepatitis B virus as a public health threat by 2030.^17^ In 2018, the WHO Western Pacific Region developed a regional framework on triple elimination, with bold policy recommendations and patient-level outcome targets.^19^ Similarly, the Pan American Health Organization has extended the triple elimination initiative to Chagas disease and other pathogens.^20^

Despite these frameworks and the advances made on triple elimination in some regions,^19^^,^^20^ progress has been slow in sub-Saharan Africa, particularly for hepatitis B virus.^21^^,^^22^ Although the African Region has committed to dual elimination of HIV and syphilis, committing to triple elimination of mother-to-child transmissions would tackle three important causes of maternal and paediatric morbidity and mortality.^23^ Triple elimination also promotes the goal to provide integrated, person-centred care within antenatal care, a key entry point to the health-care system.

## Barriers to triple elimination

While programmes aimed at preventing mother-to-child transmission of HIV are well established and have had remarkable success in sub-Saharan Africa, the global target of 95% reduction in mother-to-child transmission of HIV by 2020 was not reached.^1^^,^^16^ In 2019, an estimated 83% of pregnant women infected with HIV received antiretroviral therapy. However, coverage in west and central Africa has stagnated since 2014 and was estimated to have reached only 60% in 2019.^1^ Globally, 150 000 children aged 0 to 14 years were newly infected with HIV in 2019, of which 120 000 were in sub-Saharan Africa, and 87 000 children died of HIV-related causes.^1^

In 2016, over 600 000 cases of congenital syphilis globally were estimated to have occurred, which resulted in 355 000 preventable adverse birth outcomes, including 204 000 infant deaths.^2^ Two thirds of congenital syphilis cases were in the African Region where the prevalence of maternal syphilis was 1.5% (564 000/37 150 000).^2^ Most infants with congenital syphilis were born to women who attended antenatal care but were not screened or treated for syphilis. Fewer than half of pregnant women were screened for syphilis in the African Region in 2016 and of those testing positive, an estimated 76% were treated.^2^

Chronic hepatitis B infection is a main cause of morbidity and mortality globally due to chronic liver disease and liver cancer. Although hepatitis B can be prevented through hepatitis B virus vaccination, in 2015, 257 million people in the world were positive for HBsAg. WHO estimated that hepatitis B caused about 900 000 deaths worldwide and 90 000 deaths in sub-Saharan Africa from liver cirrhosis or cancer.^3^ The WHO African and Western Pacific Regions are the most affected by hepatitis B, with 68% of infections. This percentage is equivalent to about 60 million people with chronic hepatitis B in sub-Saharan Africa, or a 6.1% prevalence, with countries in west and central Africa most affected.^3^

Age is the main determinant of the risk of chronic hepatitis B infection: estimates suggest that about 90% of hepatitis B infections acquired in the neonatal period become chronic, but < 5% of infections acquired in adulthood become chronic.^24^ Therefore, preventing mother-to-child transmission of hepatitis B infection is an important intervention.^25^ While the prevalence of hepatitis B virus in children younger than 5 years has decreased in most regions over the past 2 decades (from an estimated 4.3% in the 1990s before the vaccination to 1.3% in 2015, globally), the prevalence of hepatitis B virus in children younger than 5 years is still 3% in sub-Saharan Africa, and about 360 000 children are infected every year.^3^^,^^22^ The African Region is the only WHO region that has not met the target of reducing infection rates to less than 1% in children younger than 5 years.^3^ While coverage of hepatitis B virus vaccination in children has improved, timely hepatitis B virus monovalent vaccination, which can significantly reduce mother-to-child transmission, varies.^26^ Although more countries in the WHO African Region recommend the hepatitis B monovalent vaccine, estimated coverage is only 4% compared with 34–84% for other WHO regions.^12^^,^^27^ Even where the vaccine is available, coverage is not optimal; for example, estimates of coverage are 63% in Senegal and 76% in Namibia, and timely vaccination in all countries in sub-Saharan Africa that have adopted vaccination policies remains below 80%.^27^ Furthermore, antenatal HBsAg testing and use of antiviral prophylaxis for mothers with hepatitis B is lacking.^26^

The continued burden of these three infections in mothers and infants in sub-Saharan Africa is linked to the lack of policies, strategies and resources to support the uptake of well established preventive and treatment interventions. Political support for elimination of mother-to-child transmission of syphilis and hepatitis B virus lags behind that for HIV. A 2017 survey of viral hepatitis policies in Member States of the WHO African Region, including 27 sub-Saharan African countries, found only 37% (10/27) of countries had a strategic and technical advisory group on viral hepatitis, 19% (5/27) had published strategic plans and 22% (6/27) had dedicated funding for viral hepatitis.^21^ WHO data from 2018 show that only 23% (11/47) of countries in the WHO African Region had adopted policies supporting the introduction of the hepatitis B virus vaccine.^27^ Similarly, six sub-Saharan African countries identified lack of political and financial support among the main reasons for poor coverage of syphilis interventions for pregnant women.^28^

Even when diagnostics and drugs are available in the country, shortages, particularly of rapid diagnostic tests and benzathine penicillin, hinder access to treatment for pregnant women.^29^ In some low- and middle-income countries, people pay themselves for antenatal care screening and treatment of syphilis or hepatitis B virus.^30^

## Integrated approach

Successful triple elimination programmes require an integrated, multidisciplinary approach, but programmes are often run separately with few opportunities and incentives for joint planning and integration of services across relevant departments of the health ministry.^13^^,^^14^ Uneven funding for the three diseases, with less investment for syphilis and hepatitis B virus compared with heavily donor-supported HIV,^31^ impedes effective integrated care for the three diseases, including provision of person-centred care. Differences in funding, political will and integration have meant that more progress has been made in controlling HIV infection than syphilis and hepatitis B virus.^32^^,^^33^

The lack of an integrated approach leads to gaps in basic delivery of care. Although most women are offered HIV testing at antenatal care, this is not true for syphilis or hepatitis B virus testing.^2^^,^^26^^,^^34^ A recent assessment of 10 sub-Saharan African countries showed a gap between HIV and syphilis screening coverage in pregnant women of 5–52%.^35^ A survey of health workers and programme managers in low- and middle-income countries found that screening for hepatitis B virus was erratic, even for pregnant women.^36^

Facility-based models of care may not provide the best access to time-sensitive interventions, such as screening. Of 20 sub-Saharan African countries with data available since 2015 on coverage of four or more antenatal care visits per pregnant woman (Angola, Benin, Burkina Faso, Burundi, Chad, Congo, Côte d’Ivoire, Ethiopia, Guinea, Malawi, Mauritania, Mozambique, Niger, Nigeria, Rwanda, Sierra Leone, South Africa, Uganda, United Republic of Tanzania and Zimbabwe), none had coverage over 80% and seven countries had coverage of less than 50%.^37^ Hepatitis B virus vaccination coverage is directly correlated with rates of births taking place in a health facility or births attended by skilled birth attendants.^38^ In sub-Saharan Africa, despite recent improvement, only 24.6% (173 885/708 143) of women delivered their babies in a health facility. Only an estimated 59% of births were attended by a skilled birth attendant in west and central Africa and 63% in eastern and southern Africa.^37^^,^^39^

Integrated monitoring and evaluation tools to track triple elimination services and outcomes are often absent or not well implemented, which complicates the tracking of interventions and outcomes for triple elimination. These limitations compromise the success of individual disease programmes and the opportunity for greater efficiency within an integrated approach to triple elimination of mother-to-child transmission.

## Achieving triple elimination

Despite the barriers described, growing political will and technical advances offer the potential for significant progress towards triple elimination in sub-Saharan Africa (Box 1 and Box 2). WHO recommends routine screening of all pregnant women for HIV, syphilis and hepatitis B.^40^^–^^42^ The Organization recently released guidance on the use of antiviral prophylaxis in women infected with hepatitis B infection who had HBeAg or high hepatitis B virus DNA load (> 200 000 IU/mL) and is working to include hepatitis B virus in its global programme for validation of the elimination of mother-to-child transmission of HIV and syphilis.^40^ These initiatives provide clear guidance to reach triple elimination and encourage action by showing that elimination of mother-to-child transmission of HIV, syphilis and hepatitis B virus is an achievable global priority. While the WHO regions in the Americas and Western Pacific have committed to elimination and validation, the WHO African Region has yet to make triple elimination validation a priority.^19^^,^^20^^,^^22^

Box 1 **Strategies to achieve triple elimination in sub-Saharan Africa**

Implement new recommendations to scale up integrated screening for maternal infections of HIV, syphilis and hepatitis B within antenatal care to guide treatment and prophylaxis interventions for mother and child.

Strengthen the establishment and increase of coverage of hepatitis B virus monovalent vaccination in sub-Saharan Africa to support the elimination of mother-to-child transmission of hepatitis B virus by 2030.

Encourage collaboration between health ministry departments and technical working groups to create an integrated, patient-centred national agenda for triple elimination, with bold targets and an appropriately resourced plan.

Empower facility and district health teams to adapt guidelines and interventions to the local context. The similarity in prevention interventions at the facility level offers a platform to integrate the prevention of mother-to-child transmission of HIV, syphilis and hepatitis B into a one-stop model.

Prioritize the development or update of national goals, policies and guidelines that are consistent with WHO guidance and strategies on the elimination of mother-to-child transmission. In particular, the recent WHO guidelines on prevention of mother-to-child transmission of hepatitis B virus provide an opportunity to create strong national guidelines for maternal antiviral treatment and prophylaxis and newborn hepatitis B vaccination.

Use potential integrated funding through the Global Fund to Fight AIDS, Tuberculosis and Malaria and hepatitis B-specific opportunities through Gavi, the Vaccine Alliance. At the same time, domestic funding can be mobilized for both rapid action and sustainability. Sustainable financing mechanisms will also be needed to eliminate user fees for the care and treatment of mothers and their sexual partners for the three diseases.

Recognize that increased and predictable demand for essential products may also lead to a more consistent and reliable supply and lower costs.

Ensure community involvement to achieve triple elimination. Without strong community participation, demand and uptake may remain low, acceptability will be compromised and important input to ensure acceptable programming will be missed.

HIV: human immunodeficiency virus; WHO: World Health Organization.

Box 2 **Key interventions to achieve triple elimination of HIV, hepatitis B and syphilis in sub-Saharan Africa**

**Screening and diagnostics**

Use HIV retesting during pregnancy and breastfeeding in high-burden settings.

Use point-of-care test to determine viral load for pregnant and breastfeeding women for rapid treatment and antiviral prophylaxis for infants.

Update screening algorithms and implement dual tests for HIV and syphilis as assay 1.

Increase access to hepatitis B surface antigen screening in antenatal care.

Incorporate new WHO diagnostic algorithms to determine eligibility for hepatitis B virus treatment.

**Prevention and treatment**

Rapidly move to improved and/or more effective antiretroviral drug regimens (e.g. dolutegravir).

Support syphilis treatment of partners and exposed infants whose mothers have not had adequate treatment.

Provide access to tenofovir-based hepatitis B treatment for eligible individuals according to WHO guidelines.

Provide universal and timely hepatitis B vaccine dose at birth followed by two or three doses at least 4 weeks apart to complete the primary course.

Ensure adequate supply of benzathine penicillin for treatment of maternal syphilis.

**Integrated and innovative models of care and logistics**

Support different service delivery models for pregnant and breastfeeding women to improve retention in care.

Improve data management and stock taking to reduce shortages of medicines and commodities.

Integrate HIV, syphilis and hepatitis B screening, prophylaxis and treatment as a core part of the antenatal care package.

HIV: human immunodeficiency virus; WHO: World Health Organization.

Global donors are supporting programmatic advances. Gavi, the Vaccine Alliance, plans to provide support for the introduction of the hepatitis B virus monovalent vaccine, which could prevent 0.3–1.2 million perinatal hepatitis B virus infections from 2021 to 2035.^43^ The Global Fund to Fight AIDS, Tuberculosis and Malaria’s successful Global Fund Replenishment, in combination with the Global Fund’s strategic support for resilient and sustainable health systems, offers countries an opportunity to deliver triple elimination interventions by applying for integrated funding to strengthen reproductive, maternal, neonatal and child health.

There are promising programmes that have integrated the prevention of mother-to-child transmission for HIV, syphilis and hepatitis B virus through coordinated funding and programme administration. For example, in 2010, China made use of the successful prevention programme for mother-to-child transmission of HIV at antenatal care to launch the first nationwide programme for the prevention of syphilis and hepatitis B virus mother-to-child transmission.^44^ The programme mobilized funding, used integrated protocols, trained midwives and set indicators and targets for all three diseases.^44^ A high coverage of screening and treatment was achieved without compromising the prevention of HIV mother-to-child transmission, and indeed the programme led to a reduction in HIV mother-to-child transmission from 8.1% (57/702) to 6.7% (145/2180). To ensure that the few resources available support efficient integration, donors, national programmes and other key stakeholders should come together to set an integrated agenda, including jointly supported targets for triple elimination, such as those in the WHO Western Pacific and American Regions. In regions where at least one of the three diseases is well supported by donors, integrated programmes must be sustained through pooled or flexible funding for integrated training, procurement and interventions.

Although new tools exist to advance the triple elimination agenda, uptake has been slow. For example, in 2019, WHO recommended the use of dual rapid diagnostic tests for HIV and syphilis to screen pregnant women.^45^ Since 2015, three dual rapid diagnostic tests have received WHO prequalification for quality assurance, a WHO approval that ensures unified standards of quality, safety and efficacy.^46^ Routine use of dual rapid diagnostic tests for HIV and syphilis has been shown to significantly increase coverage of syphilis screening in pregnant women; for example, national scale-up of dual testing in Uganda increased syphilis screening in antenatal care from 48% to 94%.^35^ However, few countries in sub-Saharan Africa have implemented dual HIV and syphilis rapid diagnostic testing because of challenges in updating national antenatal care HIV testing algorithms and test procurement.^28^

While much can be achieved with existing tools, the development of new products and models of care can accelerate triple elimination. These innovations include administration of hepatitis B virus vaccination at the household level by trained community health workers to increase coverage, and transport of the vaccine outside the strict cold chain to facilitate community-level administration of the vaccine. In China, use of village doctors to provide hepatitis B virus monovalent vaccine in the community outside a strict cold chain led to significantly greater coverage compared with community-based administration and storage of vaccine in the cold chain (51.8%; 86/166 versus 2.5%; 4/161, respectively).^47^ These interventions have improved coverage of hepatitis B vaccination in countries outside sub-Saharan Africa but they have not been adopted in sub-Saharan Africa.^36^^,^^38^

An integrated triple rapid diagnostic test for HIV, syphilis and hepatitis B virus would help to improve screening coverage in antenatal care settings, similar to the dual HIV and syphilis test. Several products are commercially available, but have not been formally compared for their diagnostic performance. No product has received the WHO prequalification quality assurance. Other products in development include simplified prefilled or patch-based vaccine delivery systems to support task-shifting of vaccination to community health workers. Long-acting antiretroviral therapy during breastfeeding may improve retention in care and reduce HIV transmission during this period of poor adherence and follow-up. New interventions and models of care need further evaluation on safety, efficacy, impact and cost–effectiveness. A prioritized operational research agenda is needed to guide the generation of evidence required to introduce and scale up innovations for triple elimination.

While it is beyond the scope of this paper to provide a detailed cost estimate for achieving triple elimination, several models have shown that triple elimination programmes, including the key interventions highlighted here, are highly cost-effective, with an incremental cost–effectiveness ratio of 114 United States dollars (US$) per disability-adjusted life year averted;^48^ programmes designed to prevent mother-to-child transmission for individual diseases are also cost-effective. A costing study based on Cambodian data estimated that the annual cost of triple elimination per pregnant woman would be US$ 11.87.^48^ However, these costs are dependent on the setting and the epidemiology of HIV, hepatitis B and syphilis in pregnant women. A modelling study estimated that the annual cost in all low- and middle-income countries to eliminate hepatitis B virus mother-to-child transmission would peak at US$ 3.4 billion a year, but would rapidly decline thereafter.^49^ Another model estimated that in a high-prevalence, low-coverage country the costs of scaling up an effective programme to prevent mother-to-child transmission of syphilis would be about US$ 1 per pregnancy.^50^ The costs to identify and treat pregnant women living with HIV, and provide testing and postnatal prophylaxis to infants exposed to HIV, range from US$ 350 to US$ 500 per pregnant woman infected with HIV.^51^ The world now faces a global economic downturn as a result of COVID-19 which will make resource mobilization more difficult and highlights the need to maximize efficiency through integration. As programmes invest in integrated triple elimination, it will be important to document the costs and potential efficiency gains to inform the expansion of interventions.

Community involvement is essential to mobilize resources and design effective, acceptable care models. At the facility and community level, there is a need to create demand, improve knowledge of these diseases and their treatment, and develop innovative models of care to ensure sustainable, robust uptake of interventions. From the start, the community must be involved; for example, community members’ input into programme design should be sought and their support for the implementation of interventions should be obtained. The HIV experience in sub-Saharan Africa has demonstrated that community involvement leads to high demand and uptake as well as increased funding and political focus.^52^

## Conclusion

Triple elimination is a strategic opportunity to tackle three main causes of morbidity and mortality for mothers and their infants through adoption of an integrated, person-centred approach. Sub-Saharan Africa, with its high burden of mother-to-child transmission for the three infections, would benefit from adopting strategies to promote triple elimination. National programmes in sub-Saharan Africa can accelerate the adoption of WHO policies and frameworks that serve to break down disease-specific vertical programmes, improve efficiency and support a person-centred and sustainable approach to triple elimination. Innovations may facilitate triple elimination programmes, but current tools and models of care already provide effective interventions to reach elimination. The sub-Saharan African health community can secure political support and make use of existing funding to realize the benefits of triple elimination.
